# Altered Resting Brain Functions in Patients With Irritable Bowel Syndrome: A Systematic Review

**DOI:** 10.3389/fnhum.2022.851586

**Published:** 2022-04-29

**Authors:** Zheng Yu, Li-Ying Liu, Yuan-Yuan Lai, Zi-Lei Tian, Lu Yang, Qi Zhang, Fan-Rong Liang, Si-Yi Yu, Qian-Hua Zheng

**Affiliations:** ^1^College of Medical Information and Engineering, Chengdu University of Traditional Chinese Medicine, Chengdu, China; ^2^Acupuncture and Tuina School, Chengdu University of Traditional Chinese Medicine, Chengdu, China; ^3^Chongqing Hospital of Traditional Chinese Medicine, Chongqing, China

**Keywords:** irritable bowel syndrome, neuroimaging, brain activity, fMRI, systematic review

## Abstract

**Background:**

The neural activity of irritable bowel syndrome (IBS) patients in the resting state without any intervention has not been systematically studied. The purpose of this study was to compare the resting-state brain functions of IBS patients with healthy controls (HCs).

**Methods:**

The published neuroimage studies were obtained from electronic databases including PubMed, EMBASE, PsycINFO, Web of Science Core, CNKI Database, Wanfang Database, VIP Database, and CBMdisc. Search dates were from inception to March 14th, 2022. The studies were identified by the preidentified inclusion and exclusion criteria. Two independent reviewers compiled the studies and evaluated them for quality and bias.

**Results:**

Altogether 22 fMRI studies were included in this review. The risk of bias of the included studies was generally low. The findings indicated that in IBS patients, increased or decreased brain areas were mostly associated with visceral sensations, emotional processing, and pain processing. According to brain network research, IBS may exhibit anomalies in the DMN, CEN, and emotional arousal networks. The fluctuations in emotion (anxiety, sadness) and symptoms in IBS patients were associated with alterations in the relevant brain regions.

**Conclusion:**

This study draws a preliminary conclusion that there are insufficient data to accurately distinguish the different neurological features of IBS in the resting state. Additional high-quality research undertaken by diverse geographic regions and teams is required to reach reliable results regarding resting-state changed brain regions in IBS.

## Introduction

Irritable bowel syndrome (IBS), one of the most prevalent chronic pain conditions, is defined by persistent stomach pain, as well as changes in stool shape and frequency (Ford and Talley, 2012; Holtmann et al., 2016; Ford et al., 2020). The global estimated prevalence of IBS ranged from 5 to 15% (Lovell and Ford, 2012; Quigley et al., 2012; Ford et al., 2020). Moreover, IBS significantly damages patients’ mental health and quality of life (Chang et al., 2010; Wong et al., 2013; Ford et al., 2017; Aziz et al., 2021), that IBS is more prevalent in patients with psychological co-morbidities (Ford et al., 2020). The incidence of IBS in combination with anxiety or depression has been estimated to be between 40 and 60% or higher (Dekel et al., 2013). Nevertheless, the pathophysiology of IBS is not fully understood, which has hampered IBS diagnosis and treatment.

Changes in the signals between the brain and the gut have also been linked to visceral hypersensitivity and central nervous alternation in IBS patients (Mayer, 2011; Kano et al., 2018). This brain-gut link is a complex integrated circuit that transmits information from the emotional and cognitive centers of the brain to the gastrointestinal tract *via* neurotransmitters (Gaman and Kuo, 2008). This explains why stress and psychological factors have a substantial association with IBS. Thus, understanding the brain activity related to IBS is critical for elucidating the pathophysiology.

Neuroimaging developments have contributed to the elucidation of the brain-gut relationship. In IBS, rectal stimulation revealed brain activation patterns consistent with visceral pain (Tillisch et al., 2011). It has been shown that the altered brain regions in IBS mainly engaged in processing affective and cognitive aspects of visceral sensitivity/pain (Skrobisz et al., 2019). These findings are comparable to those observed in repeatable reactions to pain in a variety of clinical conditions (Apkarian et al., 2005). The frontal orbital cortex integrates sensory signals including feeding behavior associated with the digestive tract, visceral pain, and other sensory signals that feed into the insula and anterior cingulate cortex (ACC). Ringel et al. (2006) observed that ACC activity (non-marginal ACC) was lower in IBS patients with drug abuse history than in IBS patients without drug abuse history and healthy controls (HCs). However, earlier studies using positron emission tomography (PET) showed that IBS patients had lower functional connectivity (FC) between ACC and anterior middle cingulate cortex (aMCC) activity when rectal dilatation was attempted (Ringel et al., 2003). Regardless of the distinctions, neuro-digestive brain imaging investigations enable a more precise assessment of the relationship between brain and peripheral processes, particularly the mechanisms through which psychosocial factors affect visceral brain sensitivity.

Resting-state imaging techniques have also been utilized to investigate abnormal spontaneous brain activity in IBS (O’Connor and Zeffiro, 2019), with the purpose of elucidating the brain’s inherent activities. Resting-state functional magnetic resonance imaging (rs-fMRI) is the most frequently utilized neuroimaging tool for examining pathological abnormalities in brain function associated with IBS. Consistent findings from several existing resting-state functional MRI studies indicated that patients with IBS have abnormal brain functions associated with versal sensation, psychological emotions (Aizawa et al., 2012; Hubbard et al., 2015; Ma et al., 2015). The exact neural substrate of IBS remains to be clarified due to the heterogeneity of the study methods, which makes it difficult to generalize the findings.

The present review aimed to identify the difference in brain functions between IBS patients and HCs without any intervention and provided a reference for further applications in neuroimage on IBS pathogenies.

## Materials and Methods

This review followed the Preferred Reporting Items for Systematic Reviews and Meta-analyses (PRISMA) 2020 statement (Page et al., 2021) and recommendations for neuroimaging meta-analysis (Nichols et al., 2017). This study evaluated available journal articles without regard for ethical considerations.

### Eligibility Criteria

Studies met the following inclusion or exclusion criteria were involved in the present review. We included studies: (1) peer-reviewed journal papers; (2) IBS patients as study population (no limitation of diagnosis); (3) a patient group with IBS and a HCs group were contrasts, there was no limitation of age in both groups; (4) Imaging technology for brain screening was not limited. The following studies were excluded: (1) reviews, systematic reviews, medical cases, research protocols, conference papers, animal studies, letter; (2) duplicate literature, including duplicates in English and Chinese, or studies using the same registration number/ethics number; (3) studies of brain structure (gray matter, white matter, and cortical thickness); or functional studies involving brain structures, such as FC of gray matter volumes; (4) small sample size (<10 IBS patients/HCs); (5) task-state studies.

### Search Strategies

Journal article from electronic databases included PubMed, EMBASE, PsycINFO, Web of Science Core, CNKI Database, Wanfang Database, VIP Database, and CBMdisc. Search dates from inception to March 14th, 2022. The reference published in English and Chinese were included. The search strategies were based on various combinations of the search terms, including “Irritable Bowel Syndrome,” “Magnetic Resonance Imaging,” “Functional MRI,” “Computed Tomography,” “Positron-Emission Tomography,” etc. Specific search strategies were appended in the Supplementary Material.

### Data Selection and Collection

L-YL searched the literature in the appropriate search library based on the search formula. Two reviewers (Y-YL and LY) independently screened the included records to complete the literature selection. Next, L-YL determined the disagreement between individual judgments. Purpose, imaging methods, diagnoses, subject characteristics, brain regions, and clinical outcomes were extracted from the study documents. The extraction operation was performed independently by two reviewers (Y-YL and LY). L-YL determined the disagreement between individual judgments. Researchers were contacted by L-YL *via* email for unreported data or other details. All the above operations were done in Excel.

### Risk of Bias Assessment

We assessed the study quality and risk of bias based on Nichol et al.’s (2017) study. The estimation items included: (1) research objective; (2) recruitment; (3) eligible criteria; (4) population demographics; (5) imaging methodology; (6) comparison group. Full reporting of the above six items represents a low risk of bias in this study; studies meeting the five items are at medium risk of bias; studies meeting four or lower above items are at high risk of bias.

## Results

**FIGURE 1:**
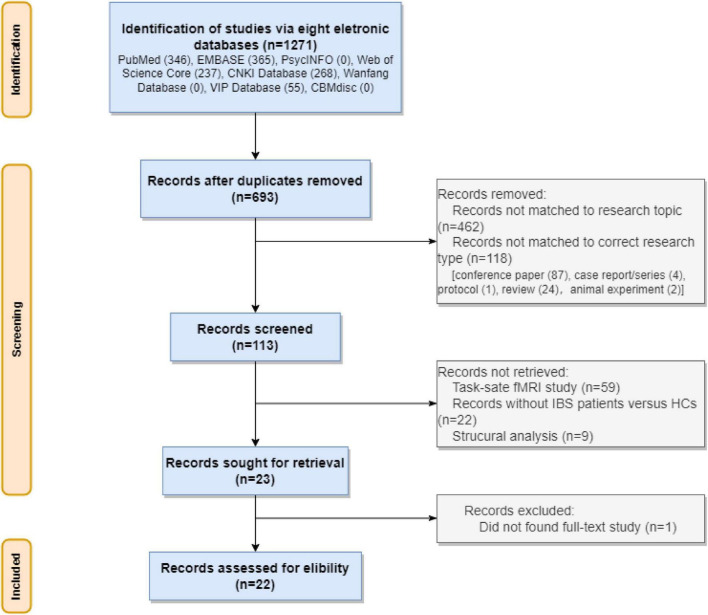
The flow diagram of the review.

A total of 1271 studies were obtained throughout the electronic databases. Of this, 578 duplicated papers were removed. After examining the research titles, we removed 462 irrelevant studies and 118 studies with inappropriate study types (87 conference papers, 4 case reports/series, 1 protocol, 24 reviews, and 2 animal studies). Of the remaining 113 studies, we removed 59 task-state fMRI studies, 21 studies with the wrong comparison, 9 studies for brain structural analysis or functional analysis based on brain structure. Of the 23 potential studies, the full text of one study (Bernstein et al., 1999) was not available. We tried to contact the corresponding author for full text but did not receive any answer. Finally, we involved 22 rs-fMRI studies. See Figure 1.

### Study Characteristics

This systematic review comprised 22 rs-fMRI studies aimed at characterizing brain function (Li et al., 2013, 2018, 2021; Zeng et al., 2013; Ma et al., 2014, 2015; Gui et al., 2015; Ke et al., 2015; Qi et al., 2015, 2016a,b; Weng et al., 2016; Qin et al., 2017; Wang et al., 2017; Nan et al., 2019a,b, 2021; Ao et al., 2021; Chen et al., 2021; Geng et al., 2021; Liu et al., 2021). These studies were conducted at a variety of research sites around China and contained a sample size of at least 10 participants. The diagnostic criteria for IBS in the included studies were respectively ROME III (Longstreth et al., 2006) in 19 studies (Li et al., 2013, 2018, 2021; Zeng et al., 2013; Ma et al., 2014, 2015; Gui et al., 2015; Ke et al., 2015; Qi et al., 2015, 2016a,b; Weng et al., 2016; Qin et al., 2017; Wang et al., 2017; Nan et al., 2019a,b; Ao et al., 2021; Chen et al., 2021), ROME IV (Drossman, 2016) in one study (Geng et al., 2021), and undefined criteria in one study (Nan et al., 2021). For IBS categories, five studies included IBS-Diarrhea (IBS-D) patients (Ke et al., 2015; Wang et al., 2017; Ao et al., 2021; Chen et al., 2021; Geng et al., 2021), and one study (Liu et al., 2021) included patients with different IBS categories, including IBS-constipation (IBS-C), IBS-mix (IBS-M) and IBS-D. Two studies by Li J’s team explored IBS with or without depression versus HCs (Li et al., 2018, 2021). Table 1 summarized the study’s characteristics.

**TABLE 1:** Study characteristics of included studies.

Author year	Country	Imaging technique	Analyze methods	Sample size (IBS/HCs)	Sex in IBS (Male/Female)	Diagnosis of IBS	Classification (n)	Age [IBS vs. HCs] (Mean ± SD)	Comparison
Zeng et al., 2013	China	rs-fMRI	ReHo	13/13	5/8	ROME III	NA	NA	IBS vs. HCs
Li et al., 2013	China	rs-fMRI	FC	21/21	14/7	ROME III	NA	IBS (41.8 ± 11.9) vs. HCs (35.9 ± 14.8)	IBS vs. HCs
Ma et al., 2014	China	rs-fMRI	fALFF	21/21	14/7	ROME III	NA	IBS (41.82 ± 11.92) vs. HCs (35.91 ± 14.76)	IBS vs. HCs
Gui et al., 2015	China	rs-fMRI	DC	21/21	14/7	ROME III	NA	IBS (41.82 ± 11.92) vs. HCs (35.91 ± 14.76)	IBS vs. HCs; pre-post (IBS)
Ke et al., 2015	China	rs-fMRI	ReHo	31/32	25/6	ROME III	IBS-D (31)	IBS (29.2 ± 9.7) vs. HCs (27.5 ± 8.6)	IBS vs. HCs
Ma et al., 2015	China	rs-fMRI	ALFF, FC	21/21	14/7	ROME III	NA	IBS (41.82 ± 11.92) vs. HCs (35.91 ± 14.76)	IBS vs. HCs
Qi et al., 2015	China	rs-fMRI	FC	31/32	25/6	ROME III	NA	IBS (29.23 ± 9.69) vs. HCs (27.47 ± 8.64)	IBS vs. HCs
Qi et al., 2016c	China	rs-fMRI	ALFF, FC	30/31	24/6	ROME III	NA	IBS (28.93 ± 9.71) vs. HCs (26.87 ± 8.08)	IBS vs. HCs
Qi et al., 2016a	China	rs-fMRI	FC	31/32	25/6	ROME III	NA	IBS (29.23 ± 9.69) vs. HCs (27.47 ± 8.64)	IBS vs. HCs
Qi et al., 2016b	China	rs-fMRI; dti	VMHC	65/67	51/16	ROME III	NA	IBS (34.00 ± 11.82) vs. HCs (31.21 ± 10.70)	IBS vs. HCs
Weng et al., 2016	China	rs-fMRI	Long-range FCD, short-range FCD, FC	31/32	25/6	ROME III	NA	IBS (27.47 ± 8.64) vs. HCs (29.23 ± 9.69)	IBS vs. HCs
Qin et al., 2017	China	rs-fMRI	ReHo	23/23	10/13	ROME III	NA	IBS (43.27 ± 7.913) vs. HCs (47.09 ± 8.837)	IBS vs. HCs
Wang et al., 2017	China	rs-fMRI	ALFF	31/20	17/14	ROME III	IBS-D (31)	IBS (25.5 ± 3.7) vs. HCs (26.1 ± 3.1)	IBS vs. HCs
Li et al., 2018	China	rs-fMRI	ReHo	35/36	20/15	ROME III	NA	DEP-IBS (37 ± 8) vs. nDEP-IBS (34 ± 11) vs. HCs (33 ± 8)	DEP-IBS vs. nDEP-IBS vs. HCs
Nan et al., 2019b	China	rs-fMRI	ReHo, ApEn	54/54	27/27	ROME III	NA	IBS (22 ± 1) vs. HCs (22 ± 1)	IBS vs. HCs
Nan et al., 2019a	China	rs-fMRI	ReHo, FC	46/60	12/34	ROME III	NA	IBS (22.02 ± 1.94) vs. HCs (22.35 ± 1.10)	IBS vs. HCs
Geng et al., 2021	China	rs-fMRI	FC	25/25	14/11	ROME IV	IBS-D (25)	IBS (40.5 ± 10.5) vs. HCs (40.6 ± 13.0)	IBS vs. HCs; pre-post (IBS)
Nan et al., 2021	China	rs-fMRI	Dynamic FC	46/61	34/12	NA	NA	IBS (22.0217 ± 1.9378) vs. HCs (22.3279 ± 1.0912)	IBS vs. HCs
Ao et al., 2021	China	rs-fMRI	fALFF	13/14	8/5	ROME III	IBS-D (13)	IBS (32.23 ± 5.96) vs. HCs (29.14 ± 5.92)	IBS vs. HCs
Chen et al., 2021	China	rs-fMRI	ALFF, ReHo, FC	36/36	16/20	ROME III	IBS-D (36)	IBS (34.36 ± 9.53) vs. HCs (31.67 ± 8.85)	IBS vs. HCs
Li et al., 2021	China	rs-fMRI	DC	28 (nDEP-IBS), 21 (DEP-IBS)/36	17/11 (nDEP-IBS); 12/9 (DEP-IBS)	ROME III	NA	DEP-IBS (36.36 ± 7.31) vs. nDEP-IBS (32.29 ± 9.96) vs. HCs (31.67 ± 8.85)	DEP-IBS vs. nDEP-IBS vs. HCs
Liu et al., 2021	China	rs-fMRI; dti	VMHC	34/33	18/16	ROME IV	IBS-D (24); IBS-C(4); IBS-M (6)	IBS (27.35 ± 4.40) vs. HCs (25.67 ± 4.56)	IBS vs. HCs

*rs-fMRI, resting-state functional magnetic resonance; dti, diffusion tensor imaging; ReHo, regional homogeneity; FC, functional connectivity; ALFF, amplitude of low frequency fluctuation; DC, degree centrality; VMHC, voxel-mirrored homotopic connectivity; FCD, functional connectivity density; ApEn, Voxel- based approximate entropy; IBS, irritable bowel syndrome; IBS-D, irritable bowel syndrome patients with diarrhea; IBS-C, irritable bowel syndrome patients with constipation; IBS-M, mixed irritable bowel syndrome patients; HCs, health controls; pre-post, post-treatment versus pre-treatment; DEP, depressive; nDEP, non-depressive.*

### Study Quality and Risk of Bias

Two reviewers (Y-YL and LY) independently evaluated the risk of bias through six items and eventually reached a consensus. Among all, 16 studies were low risk of bias (Gui et al., 2015; Ke et al., 2015; Ma et al., 2015; Qi et al., 2015, 2016a,b; Weng et al., 2016; Qin et al., 2017; Li et al., 2018, 2021; Nan et al., 2019a; Ao et al., 2021; Chen et al., 2021; Geng et al., 2021; Liu et al., 2021). Four studies reduced to medium risk of bias, for undetailed exclusion criteria (Li et al., 2013; Ma et al., 2014; Nan et al., 2021), or insufficient baseline (Wang et al., 2017). In addition, two studies were considered high risk of bias (Zeng et al., 2013; Nan et al., 2019b). One study lacked the descriptions of exclusion criteria and population demographics (Zeng et al., 2013). Another study made no reference to specific recruitment or eligibility criteria (Nan et al., 2019b). See Table 2.

**TABLE 2:** The quality and risk of bias for studies.

Author year	Research objectives	Recruitment	Eligible criteria	Population demographics	Imaging methodology	Comparison group	Risk of bias
Zeng et al., 2013	Y	Y	N	N	Y	IBS vs. HCs	High
Li et al., 2013	Y	Y	N	Y	Y	IBS vs. HCs	Medium
Ma et al., 2014	Y	Y	N	Y	Y	IBS vs. HCs	Medium
Gui et al., 2015	Y	Y	Y	Y	Y	IBS vs. HCs; pre-post (IBS)	Low
Ke et al., 2015	Y	Y	Y	Y	Y	IBS vs. HCs	Low
Ma et al., 2015	Y	Y	Y	Y	Y	IBS vs. HCs	Low
Qi et al., 2015	Y	Y	Y	Y	Y	IBS vs. HCs	Low
Qi et al., 2016c	Y	Y	Y	Y	Y	IBS vs. HCs	Low
Qi et al., 2016a	Y	Y	Y	Y	Y	IBS vs. HCs	Low
Qi et al., 2016b	Y	Y	Y	Y	Y	IBS vs. HCs	Low
Weng et al., 2016	Y	Y	Y	Y	Y	IBS vs. HCs	Low
Qin et al., 2017	Y	Y	Y	Y	Y	IBS vs. HCs	Low
Wang et al., 2017	Y	Y	Y	N	Y	IBS vs. HCs	Medium
Li et al., 2018	Y	Y	Y	Y	Y	IBS with depression vs. IBS without depression vs. HCs	Low
Nan et al., 2019b	Y	N	N	Y	Y	IBS vs. HCs	High
Nan et al., 2019a	Y	Y	Y	Y	Y	IBS vs. HCs	Low
Geng et al., 2021	Y	Y	Y	Y	Y	IBS vs. HCs; pre-post (IBS)	Low
Nan et al., 2021	Y	Y	N	Y	Y	IBS vs. HCs	Medium
Ao et al., 2021	Y	Y	Y	Y	Y	IBS vs. HCs	Low
Chen et al., 2021	Y	Y	Y	Y	Y	IBS vs. HCs	Low
Li et al., 2021	Y	Y	Y	Y	Y	IBS with depression vs. IBS without depression vs. HCs	Low
Liu et al., 2021	Y	Y	Y	Y	Y	IBS vs. HCs	Low

*IBS, irritable bowel syndrome; HCs, health controls; pre-post, post-treatment versus pre-treatment.*

### fMRI Findings

**FIGURE 2:**
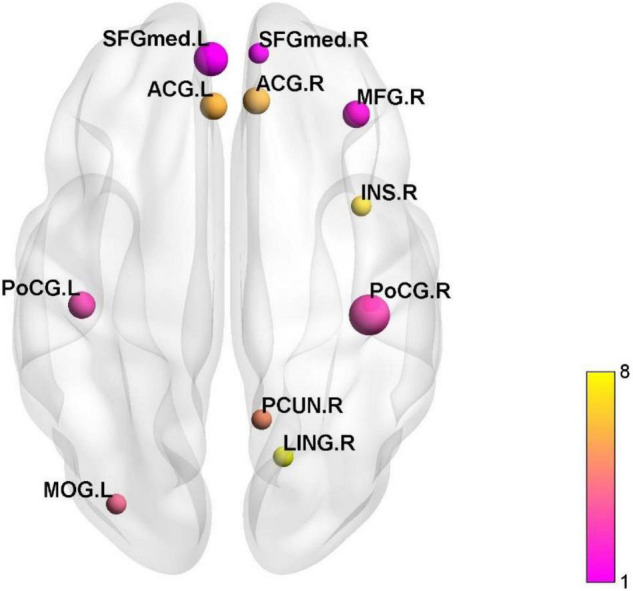
The active and inactive brain regions in the cluster analysis. SFGmed, superior frontal gyrus, medial; ACG, anterior cingulate and paracingulate gyri; MFG, middle frontal gyrus; INS, insula; PoCG, postcentral gyrus; PCUN, precuneus; LING lingual gyrus; MOG, middle occipital gyrus.

Regional homogeneity (ReHo), amplitude of low frequency fluctuations (ALFF)/fractional ALFF (fALFF), FC, dynamic FC, density connectivity (DC), functional connectivity density (FCD), voxel-mirrored homotopic connectivity (VMHC) were applied in altogether 22 studies. We did not assess meta-analysis due to the high heterogeneity between studies. Likewise, we cautioned to interpretate the outcomes. See Figure 2.

#### Amplitude of Low Frequency Fluctuations/fractional ALFF/Regional Homogeneity

We summarized 12 studies adopted ALFF (Ma et al., 2015; Qi et al., 2016c; Wang et al., 2017; Chen et al., 2021), fALFF (Ma et al., 2014; Ao et al., 2021), ReHo (Zeng et al., 2013; Ke et al., 2015; Qin et al., 2017; Li et al., 2018; Nan et al., 2019a,b), and performed clustering analysis of activated or inactivated brain regions. Nevertheless, we discovered a wide distribution of active or inactive brain regions in the 12 studies. The cluster analysis revealed that postcentral gyrus (PoCG), superior frontal gyrus (SFG), middle frontal gyrus (MFG), ACC, middle occipital gyrus (MOG), precuneus (PCUN), insula (INS), and lingual gyrus (LING) were the most frequently reported (see Figure 1). In IBS patients, activated or inactive brain areas were primarily associated with visceral sensation (PoCG, ACC, INS), emotional processing [hippocampus (HIP)], and pain processing [frontal lobe, parietal lobe, supplementary motor area (SMA), hypothalamus, HIP, cerebellum, caudate nucleus, and PCUN]. See Supplementary Table 1.

#### Functional Connectivity Density/Density Connectivity

Three studies assessed brain function in IBS patients using a data-driven FCD/DC algorithm (Gui et al., 2015; Weng et al., 2016; Li et al., 2021). Reduced DC was observed in regions of the brain associated with higher cognitive functions (prefrontal gyrus, PCUN), DMN [right orbitofrontal cortex], and middle temporal gyrus (MTG) in Gui et al.’s (2015) study. Weng et al.’s (2016) study showed reduced long-range FCD and short-range FCD in anterior MCC and inferior parietal cortex (IPC), and increased long-range FCD and short-range FCD in primary motor cortex (PoCG and PreCG). Li et al. (2021) found that differential brain activities between IBS patients and HCs was predominantly in the left medial PFC.

#### Functional Connectivity

Altogether, nine studies examined the brain regions that differed between IBS and HCs (Li et al., 2013; Ma et al., 2015; Qi et al., 2015, 2016a; Weng et al., 2016; Nan et al., 2019b; Chen et al., 2021; Geng et al., 2021). Seven studies performed voxel-wise FC analysis (Li et al., 2013; Ma et al., 2015; Qi et al., 2016a; Weng et al., 2016; Nan et al., 2019b; Chen et al., 2021; Geng et al., 2021), while two studies conducted ROI-wise analysis (Qi et al., 2015, 2016c). Regarding ROI selection, we found that the ROI was primarily located in primary sensory cortex including PoCG, emotional arousal network including amygdala (AMYG) and default mode network (DMN) including HIP. Alternatively, four studies explored the FC between regions activated or inactivated in the IBS brain (Ma et al., 2015; Qi et al., 2016c; Weng et al., 2016; Chen et al., 2021).

Nan et al. (2019a) revealed that brain activities of PoCG in IBS patients showed closely correlated with visceral pain. When classifying the IBS and the HCs, PoCG demonstrated greater sensitivity than INS. Qi et al. (2016b) found that IBS patients had higher positive resting-state FC between the AMYG and INS, midbrain, parahippocampal gyrus, precentral gyrus (PreCG), PoCG and SMA. Three studies identified HIP as the region of interest (ROI), but the FC between HIP and other brain regions varied significantly (Li et al., 2013; Qi et al., 2016c; Geng et al., 2021). The FC between HIP and brain regions associated with advanced cognition [left MFG, left superior parietal gyrus (SPG), and right PCUN] was found to be increased (Li et al., 2013). The findings of Geng et al.’s (2021) study showed reduced FC between emotion-related regions [bilateral inferior temporal gyrus (ITG), bilateral cingulate gyrus] and HIP of IBS-D patients with mood disturbances. However, in the Ma et al.’s (2015) study, no FC were identified between HIP and other brain regions. Furthermore, Qi et al. (2016c) demonstrated that IBS patients had lower FC across DMN subregions, most notably between the PCUN and ACC, and prefrontal cortex (PFC).

#### Voxel-Mirrored Homotopic Connectivity

Two studies (Qi et al., 2016b; Liu et al., 2021) found increased VMHC in the cuneus, occipital, and posterior cingulate gyrus in patients with IBS, suggesting increased strength of inter-regional temporal correlations or functional connectivity between the cerebral hemispheres.

#### Brain Activity Associated With Clinical Characteristics

Associations between clinical characteristics and the brain activity were explored in 12 studies (Zeng et al., 2013; Ke et al., 2015; Qi et al., 2015, 2016a,b; Weng et al., 2016; Li et al., 2018, 2021; Ao et al., 2021; Chen et al., 2021; Liu et al., 2021). The following rating scales were used to assess clinical symptoms for IBS patients: anxiety (Hamilton Anxiety Scale, HAMA scale), depression (Hamilton Depression Scale, HAMD scale), IBS symptom severity (IBS -symptom severity system, IBS-SSS), pain intensity of IBS (visual analog scale, VAS), IBS duration, gastrointestinal symptoms (gastrointestinal symptom rating scale, GSRS), quality of life (IBS-quality of life, IBS-QOL). The result showed that brain activities in SFG (Zeng et al., 2013), SMA (Zeng et al., 2013), midbrain (Chen et al., 2021) were positively connected with anxiety of IBS, and that in PCC (posterior cingulate cortex) (Li et al., 2018), midbrain (Chen et al., 2021), INS (Li et al., 2021) were positively connected with depression of IBS. The IBS-SSS scores positively or negatively correlated with FCD/FC/ReHo values in certain brain regions. Qi et al.’s (2016a) and Weng et al.’s (2016) study showed that the functional activities of INS were positively correlated with IBS symptoms. Qi et al. (2015) showed that the average DMN FC was negatively correlated with IBS-SSS in IBS patients. Otherwise, Ke et al. (2015) found that the IBS-SSS scores positively correlated with ReHo values in the left thalamus (THA) and negatively correlated with those in the right ventral medial PFC and MFG. Two studies showed that GSRS scores were positively correlated activities of midbrain and PoCG (Li et al., 2018; Chen et al., 2021).

Ke et al. (2015) found that the ReHo values of the PFC was found to be negatively correlated with the pain intensity. Qi et al. (2016c) also found the negative FC between medial PFC and cuneus was negatively correlated with the patients’ pain intensity. Three studies demonstrated that the disease duration and brain activity in brain regions associated with pain processing and perceptions (caudate, PCUN, SMA, INS, MCC) were positively connected (Ke et al., 2015; Weng et al., 2016; Ao et al., 2021).

## Discussion

### Summary of the Present Study

This study was a descriptive analysis of the 22 rs-fMRI studies examining the difference in brain activity between IBS patients and healthy controls. The included studies were scored in line with the guidance for neuroimaging meta-analysis. The majority (16/22) of imaging studies included in this review were completely stated and included all pertinent entries. In comparison to a prior high-quality systematic review (Tillisch et al., 2011), we eliminated task-state studies including rectal distension, which necessitates a different interpretation of our findings. Clustering of activated or inactive brain regions revealed that brain regions related with IBS anomalies were mostly associated with visceral feeling, emotional processing, and pain processing. The DMN, central execution network (CEN), and emotional arousal network are the study’s focal areas.

### The Increased-Decreased Brain Regions in Irritable Bowel Syndrome

The results of this study suggest decreased or increased of extensive frontal and parietal brain regions in patients with IBS, but there are no uniform conclusions. The altered brain regions in IBS patients are associated with visceral pain during resting state. When visceral pain pathways become sensitive, painful sensations persist, making this persistent chronic pain difficult to treat with classical medications (Lelic et al., 2014). Previous studies suggest that abnormal brain area changes caused by visceral pain are also present in patients with long-term IBS in remission, and not only during the rectal distension (Weaver, 2016; Saps, 2017). The insula (INS), hypothalamus (HIP), amygdala (AMYG), cingulate cortex, and prefrontal cortex (PFC) were the primary brain regions associated with pain processing (Liu et al., 2016). The results of three studies showed the inactivation of INS (Weng et al., 2016; Qin et al., 2017; Wang et al., 2017), which revealed the deterioration of pain inhibitory pathways in IBS patients (Seminowicz et al., 2010). Comparatively, the ACC and prefrontal cortex (PFC) are part of the medial pain system, which mediates pain experience, emotional and cognitive components (Bliss et al., 2016; Smith et al., 2021). The included studies also showed inactivation of ACC (Ke et al., 2015; Qi et al., 2016c) and PFC (Ke et al., 2015; Qi et al., 2016c; Weng et al., 2016; Li et al., 2021), suggesting that pain experience and cognition in IBS patients abnormalities.

### The Altered Brain Networks in Irritable Bowel Syndrome

The DMN is critical for maintaining resting brain function and is thought to be preferentially affected by chronic pain (Farmer et al., 2012). The DMN consists of highly interconnected medial PFC, ACC/PCC and bilateral inferior parietal cortex (IPC), HIP and other brain regions (Raichle, 2015). Qi et al. (2015) demonstrated that reduced FC in DMN subregions between the ACC and PCUN in IBS patients compared to healthy controls, partially explaining the dysregulation of visceral sensation (Naliboff et al., 2006; Albert et al., 2012). There is now a general consensus that HIP dysfunction in IBS patients has been presented in studies (Aizawa et al., 2012). In the present study, three studies explored FC with the hippocampus as the ROI and reported disparate results (Li et al., 2013; Ma et al., 2015; Geng et al., 2021). Li et al. (2013) found enhanced FCs between the right HIP and brain regions associated with higher cognitions (left MFG, left SPG, and right PCUN) in IBS patients. However, Ma et al. (2015) did not find significant FCS between hippocampus and ACC, and MCC. Geng et al. (2021) identified reduced right HIP-left insula FC in IBS-D patients compared to HCs, possibly due to abnormal activation in this brain region and decreased neural activity in the brain, resulting in reduced emotional regulation.

The DMN showed activation during resting waking states, while the CEN showed activation during cognitively and emotionally challenging activities. CEN maintains and manipulates information in working memory and is also responsible for decision making and problem solving in the pursuit of goal-oriented behavior. In this study, IBS patients had abnormalities in the extensive frontoparietal area (DLPFC, ACC, orbitofrontal cortex, parietal cortex). The excessive concern of IBS patients about their gastrointestinal symptoms further exacerbates visceral hypersensitivity and causes abnormalities in CEN. Emotional arousal networks play an important role in determining the magnitude and duration of autonomic regulation of various intestinal functions. The AMYG is an important link in the emotional arousal network in visceral pain. Qi et al. (2016a) found disturbed resting-state functional connectivity of the amygdala with cortical limbic regions in patients with IBS, which may partially explain the enhanced emotional arousal and visceral information processing associated with IBS.

### Psychological Factors in Irritable Bowel Syndrome

The psychiatric co-morbidity of IBS often contribute to a vicious cycle of gastrointestinal symptoms. Li et al. (2018, 2021) showed that depressed IBS patients have impaired brain function in the sensorimotor network, limbic network, suggesting anomalies in nociceptive perception in depressed individuals. From these two studies, whether brain alterations are specific to chronic visceral pain in IBS could not be determined. Therefore, studies on psychological aspects in IBS should be expanded to focus on trait factors (anxiety, depression) or state factors (poor mood) to discover whether brain abnormalities are specific to chronic visceral pain in IBS (Mayer et al., 2019).

## Summary and Conclusion

In this retrospective review, rs-fMRI studies with different analytical methods were summarized. These findings pave the way for further investigation of the brain-gut axis pathways in IBS. However, the widespread increased/decreased brain regions in IBS remains unexplained. There is still a need for: more directed, high-quality studies to explore (1) objective brain biomarkers of IBS. (2) central-peripheral functional links in IBS (3) psychological modulation of central pain processing in IBS.

## Author Contributions

ZY and L-YL: concept and design. L-YL, Y-YL, and LY: acquisition of data. ZY, L-YL, and Z-LT: drafting of the manuscript. QZ, F-RL, Q-HZ, and S-YY: critical revision of the manuscript. All authors contributed to the article and approved the submitted version.

## Conflict of Interest

The authors declare that the research was conducted in the absence of any commercial or financial relationships that could be construed as a potential conflict of interest.

## Publisher’s Note

All claims expressed in this article are solely those of the authors and do not necessarily represent those of their affiliated organizations, or those of the publisher, the editors and the reviewers. Any product that may be evaluated in this article, or claim that may be made by its manufacturer, is not guaranteed or endorsed by the publisher.

## Abbreviations

ACC: anterior cingulate cortex
ALFF: amplitude of low frequency fluctuations
AMYG: amygdala
CEN: central execution network
DC: density connectivity
DMN: default mode network
FC: functional connectivity
FCD: functional connectivity density
GSRS: gastrointestinal symptom rating scale
HAMA scale: Hamilton Anxiety Scale
HAMD scale: Hamilton Depression Scale
HCs: healthy controls
HIP: hippocampus
IBS: irritable bowel syndrome
IBS-D: IBS-Diarrhea
IBS-C: IBS-constipation (IBS-C)
IBS-M: IBS-mix
IBS-QOL: irritable bowel syndrome-quality of life
IBS-SSS: irritable bowel syndrome-symptom severity system
INS: insula
IPC: inferior parietal cortex
ITG: inferior temporal gyrus
LING: lingual gyrus
MCC: middle cingulate cortex
MFG: middle frontal gyrus
MOG: middle occipital gyrus
PCC: posterior cingulate cortex
PCUN: precuneus
PET: positron emission tomography
PFC: prefrontal cortex
PoCG: postcentral gyrus
PreCG: precentral gyrus
PRISMA: Preferred Reporting Items for Systematic Reviews and Meta-analyses
ReHo: regional homogeneity
ROI: region of interest
rs-fMRI: resting-state functional magnetic resonance imaging
SFG: superior frontal gyrus
SMA: supplementary motor area
SPG: superior parietal gyrus
THA: thalamus
VAS: visual analog scale
VMHC: voxel-mirrored homotopic connectivity.
