# Supplementary material for: Altered Resting Brain Functions in Patients With Irritable Bowel Syndrome: A Systematic Review
Source: Front Hum Neurosci. 2022 Apr 29;16:851586. doi: 10.3389/fnhum.2022.851586 (PMC9105452; doi:10.3389/fnhum.2022.851586)
Supplement: Supplementary file 1 [file Data_Sheet_1.pdf]

Table S1. Summary of whole brain activities of the included studies.

Author Year	Analyze Method	Contrast	Brain regions
Zeng SQ, et al.(2013)	REHO	IBS>HCs	*None.*
		IBS<HCs	*Right SMA, right SFG*
Ma XF, et al.(2014)	fALFF	IBS>HCs	*Right TPOmid, PoCG, PreCG, right SFG.*
		IBS<HCs	*Cerebellum.*
Gui RH, et al.(2015)	DC	IBS>HCs	*None.*
		IBS<HCs	*Right SFG, right MTG, right MFG, right orbitofrontal cortex, right PCUN.*
Ke J, et al.(2015)	REHO	IBS>HCs	*PoCG, left SPG, right THA, calcarine, vermis.*
		IBS<HCs	*Anterior MCC/posterior ACC, subgenual ACC/ ventral medial PFC, Left dorsal medial PFC/SFG, right CAU, Right SFG, MFG, Right IFG, Left PCUN*
Ma XF, et al.(2015)	ALFF	IBS>HCs	*Left calcarine, left MCC.*
		IBS<HCs	*Left SFG, right STG, right HIP, PoCG, right MFG.*
Qi RF, et al.(2016)	ALFF	IBS>HCs	*INS, cuneus.*
		IBS<HCs	*Medial PFC, right MFG, right ORBsup, dorsal ACC, ventral ACC, PCC, IPL.*
Weng YF, et al.(2016)	long-range FCD	IBS>HC	*PreCG, PoCG, right SMA.*
		IBS<HC	*Right anterior INS, IPL, anterior MCC.*
	short-range FCD	IBS>HC	*PreCG, PoCG/PCUN, occipital lobe.*
		IBS<HC	*Ventral medial PFC/sub genual MCC, anterior MCC, caudate, right DLPFC, PoCG/PCUN, IPL.*
Qin M, et al.(2017)	REHO	IBS>HCs	*Left occipital lobe.*
		IBS<HCs	*Right INS, right STG, MTG.*
Wang LJ, et al.(2017)	ALFF	IBS>HCs	*THA, temporal lobe, inferior parietal, right cerebellum anterior lobe, vermis, IOG, left MOG.*
		IBS<HCs	*Frontal lobe, right INS.*
Li J, et al.(2018)	REHO	DEP or nDEP IBS >HCs	*Left TPOsup, left PCC/PCUN, left MOG, right IPL.*
		DEP or nDEP IBS <HCs	*Right IFG, right MOG.*
Nan JF, et al.(2019)	REHO	IBS>HCs	*left IFG, left ITG/MTG, left LING, MOG/SOG.*
		IBS<HCs	*Right ANG.*
Nan JF, et al.(2019a)	REHO	IBS>HCs	*PoCG.*
		IBS<HCs	*None.*
Ao WQ, et al.(2021)	fALFF	IBS>HCs	*Left HIP, right PCUN.*
		IBS<HCs	*Left medial SFG.*
Chen XF, et al.(2021)	ALFF	IBS>HCs	*Right cerebellum posterior lobe, right LING, right calcarine, right PoCG, right medial MFG.*
		IBS<HCs	*right supramarginal gyrus, right midbrain, left PCUN, right IPL,*

	REHO	IBS>HCs	*LING, right medial SFG, right SFG, right PoCG.*
		IBS<HCs	*Left ORBinf, right SMA.*
		DEP-IBS vs nDEP-IBS vs HCs	*Left INS，left medial PFC, left PreCG.*
Li J, et al.(2021)	DC	DEP-IBS + nDEP-IBS>HCs	*None.*
		DEP-IBS + nDEP-IBS<HCs	*Left medial PFC.*

Abbreviations: REHO, regional homogeneity; ALFF, amplitude of low frequency fluctuation; FCD, functional connectivity density; DC, degree centrality; IBS, irritable bowel syndrome; HCs, health controls; DEP, depressive; nDEP, non-depressive; SMA, supplementary motor area; SFG, superior frontal gyrus; TPOmid, orbital part of middle frontal gyrus; PoCG, postcentral gyrus; PreCG, precentral gyrus; SPG, superior parietal gyrus; THA, thalamus; MCC, midcingulate cortex; ACC, anterior cingulate cortex; PFC, prefrontal cortex; CAU, caudate nucleus; MFG, middle frontal cortex; IFG, inferior frontal gyrus; PCUN, precuneus; STG, superior temporal gyrus; HIP, hippocampus; INS, insula; ORBsup, orbital part of superior frontal gyrus; PCC, posterior cingulate cortex; IPL, inferior parietal; MTG, middle temporal gyrus; IOG, inferior occipital gyrus; MOG, middle occipital gyrus; ITG, inferior temporal gyrus; LING, lingual gyrus; SOG, superior occipital gyrus; ANG, angular gyrus; ORBinf, orbital part of inferior frontal gyrus.

Table S2. FC difference between brain regions in the included studies.

Author Year	Analyze Method	Regions of Interest	Contrast	FC Details
Li SM, et al.(2013)	Voxel-wise FC	Right HIP.	Increased FC	*Right HIP-right MCC, right HIP-right PCUN, right HIP-PreCG.*
			Decreased FC	*Right HIP-left MFG, right HIP-left SPG, right HIP-left PCUN.*
Ma XF, et al.(2015)	Voxel-wise FC	Left ACC, right MTG, right HIP.	Increased FC	*Left MCC-left SFG, right MFG-left SFG, right MFG-left PCC.*
			Decreased FC	*Right MFG-right SPG, right MFG-left REC.*
Qi RF, et al.(2015)	ROI-wise FC	DMN, PCC, medial PFC, left lateral parietal cortex	Increased FC	*None.*
			Decreased FC	*Left ACC-left PCUN (DMN), left ACC-right PCUN (DMN), right ACC-right PCUN (DMN), left ORBsup-left PCUN (DMN), right ORBsup-left PCUN (DMN), right ORBsup-right PCUN (DMN), left PCUN-right MTG (DMN), right PCUN-right MTG (DMN).*
Qi RF, et al.(2016)	ROI-wise FC	INS, cuneus, medial PFC, right MFG, right ORBsup, dorsal ACC, ventral ACC, PCC, IPL.	Increased FC	*Medial PFC-right ORBsup, ventral ACC- PCC.*
			Decreased FC	*Medial PFC-left anterior INS.*
Qi RF, et al.(2016b)	Voxel-wise FC	AMYG	Increased FC	*Left AMYG-right INS, left AMYG-midbrian, left AMYG-left PreCG/left PoCG, left AMYG-right PreCG, left AMYG-left HIP, left AMYG-SMA, right AMYG-right INS, right AMYG-midbrain, right AMYG-left HIP, right AMYG-PreCG, right AMYG-right SMA.*
			Decreased FC	*None.*
Weng YF, et al.(2016)	Voxel-wise FC	THA, temporal lobe, IPL, right cerebellum anterior lobe, vermis, IOG, left MOG, frontal lobe, right INS.	Increased FC	*Anterior MCC-right anterior INS, anterior MCC-SFG, PoCG-PreCG, PoCG-PFC.*
			Decreased FC	*Anterior MCC-PCC/PCUN, right anterior INS-PCC/PCUN, PCC/PCUN-anterior MCC, PCC/PCUN-DLPFC, PCC/PCUN-IPL, medial PFC- anterior MCC, medial PFC-anterior INS, medial PFC-DLPFC, medial PFC-IPL.*
Nan JF, et al.(2019a)	Voxel-wise FC	PoCG	Increased FC	*Left PoCG-INS, left PoCG-PreCG.*
			Decreased FC	*Right PoCG-INS, right PoCG-PreCG, right PoCG-right SMA.*
Geng H, et al.(2021)	Voxel-wise FC	HIP	Increased FC	*Left HIP-left STG.*
			Decreased FC	*Left HIP-right ITG, left HIP-left FG, left HIP-left SPG, right HIP-right FG, right HIP-left ITG, right HIP-left IOG, right HIP-left REC, right HIP-left INS, right HIP-left PCUN, right HIP-right ANG, right HIP-left MOG, right HIP-right PCUN, right HIP-left SPG.*
Chen XF, et al.(2021)	Voxel-wise FC	right LING, right SFG, right PoCG,	Increased FC	*None.*

	right IPL, right SMA, right cerebellum posterior lobe, right supramarginal gyrus, right midbrain, left ORBinf, left PCUN	Decreased FC	*None.*

Abbreviations: FC, functional connectivity; HIP, hippocampus; ACC, anterior cingulate cortex; MTG, middle temporal gyrus; DMN, default mode network; PCC, posterior cingulate cortex; PFC, prefrontal cortex; INS, insula; MFG, middle frontal gyrus; ORBsup, orbital part of superior frontal gyrus; IPL, inferior parietal; AMYG, amygdala; THA, thalamus; IOG, inferior occipital gyrus; MOG, middle occipital gyrus; PoCG, postcentral gyrus; LING, lingual gyrus; SFG, superior frontal gyrus; SMA, supplementary motor area; ORBinf, orbital part of inferior frontal gyrus; PCUN, precuneus; MCC, midcingulate cortex; PreCG, precentral gyrus; SPG, superior parietal gyrus; REC, rectus; STG, superior temporal gyrus; ITG, inferior temporal gyrus; FG, fusiform gyrus; ANG, angular gyrus; DLPFC, dorsolateral prefrontal cortex.

Table S3. Summary of brain activities associated with clinical symptoms.

Author Year	Analyze Method	Symptom (measurement)	Correlation Analysis
Zeng SQ, et al.(2013)	REHO	Anxiety (HAMA scale)	The HAMA scores of IBS patients showed positive correlations with REHO values in the right SFG and left SMA.
Ke J, et al.(2015)	REHO	IBS duration (months)	The disease duration of IBS patients showed positive correlations with REHO values in the right PoCG and negative correlations with REHO values in the right anterior MCC/SMA and bilateral INS.
		Symptoms' severity (IBS-SSS)	The IBS-SSS scores positively correlated with REHO values in the left THA and negatively correlated with those in the right ventral medial PFC and MFG.
		Pain intensity (VAS)	The pain intensity positively correlated with REHO values in the left PoCG and negatively correlated with those in the right dorsal medial PFC and left MFG.
Qi RF, et al.(2015)	FC	Symptoms' severity (IBS-SSS)	The average DMN FC was negatively correlated with IBS-SSS in IBS patients.
Qi RF, et al.(2016)	FC	Pain intensity (VAS)	The negative FC between medial PFC and cuneus was negatively correlated with the patients' pain intensity.
Qi RF, et al.(2016a)	FC	Pain intensity (VAS)	The pain intensity in IBS patients positively correlated with FC between the left AMYG and bilateral SMA, PoCG, PreCG, and INS; and the FC between the right AMYG and the right PreCG and right INS.
		Symptoms' severity (IBS-SSS)	The IBS-SSS positively correlated with FC between the left AMYG and bilateral INS and the midbrain and with FC between the right AMYG and right INS.
Qi RF, et al.(2016b)	VMHC	Quality of life (IBS-QOL)	The VMHC value in ventral ACC showed a slight negative correlation with the QOL scores of IBS patients.
Weng YF, et al.(2016)	FCD	Symptoms' severity (IBS-SSS)	Positive correlations were found between IBS-SSS scores and long-range FCD values in right anterior INS.
		IBS duration (months)	The disease duration and short-range FCD values were positively connected in left caudate.
Li J, et al.(2018)	REHO	Depression (HAMD scale)	The HAMD scores of DEP-IBS patients were positively connected with REHO values in left PCC.
		Gastrointestinal symptoms (GSRS)	The GSRS scores of DEP-IBS patients were positively connected with REHO values in left PoCG.
Ao WQ, et al.(2021)	fALFF	IBS duration (months)	The results showed a positive correlation between the duration of IBS-D and the fALFF value in the right PCUN.
Chen XF, et al.(2021)	ALFF	Anxiety (HAMA scale)	The ALFF values in the right midbrain positively correlated with HAMA scores in IBS-D patients.
		Depression (HAMD scale)	The ALFF values in the right midbrain positively correlated with HAMD scores in IBS-D patients.
		Gastrointestinal symptoms (GSRS)	The ALFF values in the right midbrain positively correlated with GSRS scores in IBS-D patients.
Li J, et al.(2021)	DC	Depression (HAMD scale)	Partial correlation analysis showed that the DC value of the left INS of DEP-IBS patients was significantly negatively correlated with HAMD scores.

Liu GY, et al.(2021)	VMHC	Symptoms' severity (IBS-SSS)	The VMHC value and the IBS-SSS were found significant negative correlation in the bilateral middle occipital gyrus.

Abbreviations: REHO, regional homogeneity; ALFF, amplitude of low frequency fluctuation; FCD, functional connectivity density; DC, degree centrality; FC, functional connectivity; voxel-mirrored homotopic connectivity, VMHC; HAMA scale, Hamilton anxiety scale; HAMD scale, Hamilton depression scale; IBS, irritable bowel syndrome; IBS-SSS, irritable bowel syndrome symptom severity system; IBS-QOL, irritable bowel syndrome quality of life; GSRS, gastrointestinal symptom rating scale; SFG, superior frontal gyrus; SMA, supplementary motor area; PoCG, postcentral gyrus; MCC, midcingulate cortex; INS, insula; THA, thalamus; PFC, prefrontal cortex; MFG, middle frontal gyrus; DMN, default mode network; AMYG, amygdala; PreCG, precentral gyrus; ACC, anterior cingulate cortex; DEP, depressive; PCC, posterior cingulate cortex; IBS-D, irritable bowel syndrome with diarrhea; PCUN, precuneus.

DDL：2022. 03. 14

Search Strategy

PubMed

Search	Query	Results
#1	(IBS[Title/Abstract]) OR (Irritable bowel syndrome[MeSH Terms])	13174
#2	(Magnetic Resonance Imaging[MeSH Terms]) OR (MRI[Title/Abstract]) OR (fMRI[Title/Abstract]) OR (Functional MRI[Title/Abstract]) OR (rs-fMRI[Title/Abstract]) OR (resting-state fMRI[Title/Abstract])	615021
#3	(Computed Tomography[MeSH Terms]) OR (Positron-Emission Tomography[MeSH Terms]) OR (CT[Title/Abstract]) OR (PET[Title/Abstract])	752809
#4	(BOLD[Title/Abstract]) OR (Blood oxygenation level dependent[Title/Abstract])	13537
#5	#1 AND (#2 OR #3 OR #4)	346

Search: ((((BOLD[Title/Abstract]) OR (Blood oxygenation level dependent[Title/Abstract])) OR ((Computed Tomography[MeSH Terms]) OR (Positron-Emission Tomography[MeSH Terms]) OR (CT[Title/Abstract]) OR (PET[Title/Abstract]))) OR ((Magnetic Resonance Imaging[MeSH Terms]) OR (MRI[Title/Abstract]) OR (fMRI[Title/Abstract]) OR (Functional MRI[Title/Abstract]) OR (rs-fMRI[Title/Abstract]) OR (resting-state fMRI[Title/Abstract]))) AND ((IBS[Title/Abstract]) OR (Irritable bowel syndrome[MeSH Terms]))

EMBASE

Embase <1980 to 2022 March 13>

1 Magnetic Resonance Imaging.ab,ti. 311454
2 MRI.ab,ti.475289
3 fMRI.ab,ti. 64232
4 Functional MRI.ab,ti. 53409
5 rs-fMRI.ab,ti. 3122
6 resting-state fMRI.ab,ti. 6882
7 Computed Tomography.ab,ti. 353080

8 Positron-Emission Tomography.ab,ti. 83633
9 CT.ab,ti. 655819
10 PET.ab,ti. 196816
11 1 or 2 or 3 or 4 or 5 or 6 or 7 or 8 or 9 or 10 1493270
12 IBS.ti. 2846
13 Irritable bowel syndrome.ti. 12247
14 12 or 13 14216
15 11 and 14 365

PsycINFO

((Title: IBS) OR (Title: Irritable bowel syndrome)) AND ((Title: PET) OR (Title: CT) OR (Title: Positron-Emission Tomography) OR (Title: Computed Tomography) OR (Title: resting-state fMRI) OR (Title: rs-fMRI) OR (Title: Functional MRI) OR (Title: fMRI) OR (Title: MRI) OR (Title: Magnetic Resonance Imaging))

Result: 0

Web of Science

1. TI=((IBS) OR (Irritable bowel syndrome))
2. TS=((PET) OR (CT) OR (Positron-Emission Tomography) OR (Computed Tomography) OR (resting-state fMRI) OR (rs-fMRI) OR (Functional MRI) OR (fMRI) OR (MRI) OR (Magnetic Resonance Imaging))

1 AND 2

Result: 233

CNKI Database

学术期刊(同义词扩展)

(SU='磁共振成像技术' OR SU='核磁共振成像' OR SU='功能性核磁共振成像' OR SU='血氧水平依赖成像' TKA='正电子发射断层摄影术' OR SU='计算机断层扫描' OR TKA='MRI' OR TKA='fMRI' OR TKA='Functional MRI' OR TKA='BOLD' OR TKA='rs-fMRI' OR TKA='resting-state fMRI' OR TKA='CT' OR TKA='PET') AND (SU='肠易激综合症' OR SU='IBS')

Result: 268

Wanfang Database

期刊论文

检索表达式(中英文扩展&主题词扩展): (题名或关键词:(磁共振成像技术) or 题名或关键词:(核磁共振成像) or 题名或关键词:(功能性核磁共振成像) or 题名或关键词:(血氧水平依赖成像) or 题名或关键词:(正电子发射断层摄影术) or 题名或关键词:(计算机断层扫描)) and (题名或关键词:(肠易激综合症))

Result: 0

VIP Database

题名或关键词=(计算机断层扫描+computed tomography+CT+计算机体层摄影+发射型正电子断层显像+正电子发射成像+正电子发射计算机体层扫描+正电子发射断层成像+正电子发射断层扫描仪+正电子发射断层扫描+正电子发射体层显像+正电子发射型计算机断层+正电子发射型计算机断层显像+正电子发射断层扫描术+正电子发射断层摄影技术+正电子发射断层显像+正电子发射计算机体层成像+正电子体层显像+正电子发射断层扫描成像+正电子发射断层摄影+正电子发射计算机断层显像+正电子发射计算机断层扫描+正电子成像+正电子显像+正电子发射型计算机断层扫描+正电子发射断层摄影术+正电子发射计算机断层成像+正电子发射体层摄影+正电子发射显像+正电子发射计算机断层+正电子发射断层扫描技术+正电子断层成像+正电子发射计算机体层摄影+正电子发射体层成像+正电子发射+断层血氧水平依赖成像+功能性核磁共振成像+磁共振成像 +磁共振成像诊断+磁共振技术+磁共振影像+磁共振+磁共振成象+磁共振成像术+核磁影像+磁共振显像+磁共振检查+磁共振扫描+磁共振影像学+磁共振成像技术+磁共振造影+核磁共振成像技术+核磁共振成像+磁振造影+核磁共振影像+x 线计算机磁共振成像显像+Magnetic Resonance Imaging +MR+MRI+fMRI+Functional MRI+rs-fMRI+resting-state fMRI) AND 题名或关键词=(肠易激综合征+irritable bowel syndrome+大肠激躁症+肠道易激综合症+肠易激惹综合征+结肠易激综合征+肠易激综合症+肠道易激综合征+肠应激综合征)

Result: 55

CBMdisc

("磁共振成像技术"[加权:扩展] OR "核磁共振成像"[加权:扩展] OR "功能性核磁共振成像"[加权:扩展] OR "血氧水平依赖成像"[加权:扩展] OR "正电子发射断层摄影术"[加权:扩展] OR "计算机断层扫描"[加权:扩展]) AND ("肠易激综合征"[加权:扩展])

Result: 0
